# The impact of policy interventions to promote the uptake of biosimilar medicines in Belgium: a nationwide interrupted time series analysis

**DOI:** 10.1186/s12961-023-01015-4

**Published:** 2023-07-06

**Authors:** Yannick Vandenplas, Steven Simoens, Philippe Van Wilder, Arnold G. Vulto, Isabelle Huys

**Affiliations:** 1 Department of Pharmaceutical and Pharmacological Sciences, KU Leuven, Leuven, Belgium; 2 Ecole de Santé Publique, Université Libre de Bruxelles (ULB), Brussels, Belgium; 3 Hospital Pharmacy, Erasmus University Medical Center, Rotterdam, The Netherlands

**Keywords:** Belgium, Biosimilar, Biological, Policy, Interrupted time series, ARIMA

## Abstract

**Background:**

The Belgian government has taken several measures to increase the uptake of biosimilars in past years. However, no formal evaluation of the impact of these measures has been made yet. This study aimed to investigate the impact of the implemented measures on biosimilar uptake.

**Methods:**

An interrupted time series analysis was performed using an autoregressive integrated moving average (ARIMA) model with the Box-Jenkins method. All data were expressed as defined daily doses (DDD) per month/quarter and obtained from the Belgian National Institute for Health and Disability Insurance (NIHDI). Three molecules were included in the analysis: etanercept (ambulatory), filgrastim (hospital), and epoetin (hospital). A significance level of 5% was used for all analyses.

**Results:**

In the ambulatory care, the effect of a financial prescriber incentive of 2019 was investigated. After this intervention, 44.504 (95% CI −61.61 to −14.812; *P* < 0.001) fewer etanercept biosimilar DDDs were dispensed monthly than expected in the absence of the intervention. Two interventions were modelled for biosimilars in the hospital setting. The first intervention of 2016 includes prescription targets for biosimilars and monitoring of hospitals on adequate tendering. The second intervention involves an information campaign on biosimilars. After the first intervention, a small decrease in quarterly epoetin biosimilar uptake of 449.820 DDD (95% CI −880.113 to −19.527; *P* = 0.05) was observed. The second intervention led to a larger increase in quarterly epoetin biosimilar uptake of 2733.692 DDD (95% CI 1648.648–3818.736; *P* < 0.001). For filgrastim, 1809.833 DDD (95% CI 1354.797–2264.869; *P* < 0.001) more biosimilars were dispensed immediately after the first intervention and 151.639 DDD (95% CI −203.128 to −100.150; *P* < 0.001) fewer biosimilars each quarter after the first intervention. An immediate and sustained increase of 700.932 DDD (95% CI 180.536–1221.328; *P* = 0.016) in quarterly biosimilar volume was observed after the second intervention. All other parameter estimates were not statistically significant.

**Conclusions:**

The results of this study suggest that the impact of past policy interventions to increase the uptake of biosimilars has been variable and limited. A holistic policy framework is required to develop a competitive and sustainable off-patent biologicals market in Belgium.

**Supplementary Information:**

The online version contains supplementary material available at 10.1186/s12961-023-01015-4.

## Background

Biosimilars are similar versions of authorized biological medicines, which means they are structurally and functionally highly similar and clinically equivalent to the reference product [1]. They can enter the market after loss of exclusivity of originator biologicals, after having shown a similar quality, safety and efficacy. Even after 15 years of clinical experience and 2 billion treatment days with biosimilar medicines in Europe, no signals of decreased efficacy or safety have been reported [2, 3]. This confirms the robust regulatory framework for biosimilars outlined by the European Medicines Agency [4]. Besides being clinically equivalent, biosimilars have several beneficial effects for national healthcare systems worldwide. Due to the competition they introduce in the market, prices are lowered and patients may have more and faster access to biologicals [5–8]. Moreover, biosimilars also create competition on other aspects besides price, such as patient friendliness, available dosages or strengths, and new routes of administration [7]. In this way, biosimilars increase the quality of care for patients while contributing to more financially sustainable healthcare systems.

In an ideal world, healthcare budgets are infinite. However, in reality, the resources to finance our healthcare systems are limited. Given the increasing expenses on pharmaceuticals in the past couple of years in a system that is mainly publicly funded, Belgium faces several challenges to maintain the financial sustainability of its high-quality healthcare system in the coming decades [9, 10]. Overall, biologicals contribute to approximately 34% of the pharmaceutical budget across Europe, and are therefore an important contributor to increasing expenses [5, 11, 12]. It is expected, with the advent of new precision medicine therapies, that this number will further increase in the near future [12]. Nonetheless, several biologicals are also set to lose their exclusivities in the coming years, creating significant savings opportunities for the Belgian healthcare system. With the largest savings potential yet ahead of us in the coming years, the importance of a competitive and sustainable off-patent biologicals market is larger than ever before [5, 12, 13]. However, this opportunity requires a well-functioning Belgian off-patent market to guarantee an economically viable situation for biosimilars. The Belgian National Institute for Health and Disability Insurance (NIHDI) has recognized this potential in their recent budget discussions by raising the need for effective measures to promote biosimilar use [14].

Despite the need for biosimilars to obtain a competitive and sustainable off-patent biologicals market, Belgium has experienced a difficult situation regarding the uptake of biosimilars since the very beginning [15, 16]. Biosimilar market shares have been low and only slowly increasing compared to other Western European countries such as Germany, the Netherlands, United Kingdom or France [11, 17, 18]. Multiple scientific papers have already outlined the challenges the Belgian off-patent biologicals market faces, pointing out the lack of trust and knowledge about biosimilars among healthcare providers and patients, a malfunctioning tendering system, the absence of tangible incentives to use biosimilars for healthcare providers and patients, prescribing shifts towards new alternatives and an overall non-coherent policy framework [16, 17, 19, 20]. The Belgian government attempted to address these issues with several stand-alone measures in the past decade [17]. These policy interventions focused on different aspects of the market and aimed to increase the usage of biosimilars, both in the hospital and ambulatory setting. The implementation of these measures shows that Belgian policymakers realized the need for a more competitive off-patent biologicals market [9, 21]. However, a formal scientific evaluation of the impact of these measures has not been conducted to date.

This study aimed to investigate the impact of past policy measures to increase the uptake of biosimilar medicines in Belgium. By better understanding the impact of existing or past interventions, Belgian policymakers can better tailor future policy interventions to the Belgian context.

## Methods

### Study design and aim

By means of a retrospective interrupted time series (ITS) design, we aimed to evaluate the impact of Belgian policy measures to increase the uptake of biosimilars. Several policy measures were introduced during the past years by Belgian policymakers to increase the uptake of biosimilar medicines. These can be split up in two distinct packages of measures, a first wave of policy interventions introduced in January 2016 and a second wave in December 2018–January 2019. These waves both include multiple measures with a focus on different healthcare settings or products (compare with Table 1) [17].

**Table 1:** Summary of two waves of policy interventions in Belgium to increase the uptake of biosimilars

Date of implementation	Policy intervention*	Focus
January 2016	Prescription target of 20% for biological naïve patients. In parallel, the government stated that transitioning from the reference to a biosimilar product is allowed and encouraged Monitoring of Belgian hospitals regarding compliance with the procurement legislation and uptake of biosimilars Circular letter on fair and competitive tendering to Belgian hospitals by the Ministry of Public Health and Social Affairs, with the aim to stimulate competitive procurement procedures for off-patent biologicals	Hospital and ambulatory Hospital Hospital
December 2018–June 2019	Information campaign on biosimilar medicines for healthcare providers and patients (December 2018) Financial incentive to stimulate the prescription of biosimilars for TNF-alpha inhibitors in the ambulatory setting (i.e. etanercept and adalimumab) (January 2019) Circular letter to Belgian hospitals to remind them to adequately apply the law on public procurement and the law on medicines (June 2019)	Hospital and ambulatory Ambulatory (only etanercept and adalimumab) Hospital

^*^The list of policy interventions is collected from Moorkens et al. [17]

### Data sources

All data were retrieved from the Belgian NIHDI. Depending on the setting in which medicines are dispensed, namely the hospitals or ambulatory care, different databases apply. For the hospital setting, data were retrieved from the DocPH database. This database covers medicine dispensing records of all reimbursed medicines in Belgian hospitals. The DocPH database includes quarterly data only. For the ambulatory care, the Farmanet database served as our data source. This database collects dispensing data of all reimbursed medicines for outpatient deliveries in Belgium. In contrast to the DocPH database, Farmanet contains monthly data. Both databases are managed nationally by NIHDI and contain all data on reimbursed medicines in Belgium.

### Outcome measures

We used the volume for each product as the main outcome variable. Volumes are expressed as defined daily doses (DDD) in accordance with the daily doses defined by the WHO. As discussed above, products in the ambulatory setting have monthly data and products in the hospital setting have quarterly data. We have conducted this study for three products: filgrastim (L03AA02), epoetin (B03XA01) and etanercept (L04AB01). Filgrastim and epoetin are used in the hospital setting and etanercept in the ambulatory setting. These products were chosen because they are introduced early enough into the Belgian market to measure the impact of past policy measures. Other relevant products with biosimilars are introduced too recently in Belgium and thus have too few data points to conduct the analysis. A list of the available biosimilar products per molecule in Belgium, along with their date of reimbursement, is provided in Additional file 1.

The study covers a period of 9 years in total, from January 2013 until June 2021 for hospital data and from January 2013 until August 2021 for outpatient data. For epoetin and filgrastim, the whole period was analysed since for both products biosimilars were launched before January 2013. For etanercept, only data after March 2017 was analysed because no biosimilar market volumes were observed before.

### Statistical analysis

An interrupted IITS analysis was conducted to examine the impact of policy interventions to simulate the uptake of biosimilars. ITS analyses are a powerful quasi-experimental method to investigate the longitudinal effect of an intervention when randomized control trials are not feasible [22]. The underlying assumption of ITS analyses is that the trend before the intervention could be extrapolated after the intervention, in case the intervention did not occur [22–24]. Therefore, ITS analyses aim to assess whether and to what extent the trend differs pre- and post-intervention. For the ITS analysis, we followed the procedure to evaluate public health interventions as described by Bernal et al. [24].

As mentioned above, two distinct waves of policy interventions to increase the uptake of biosimilars were introduced in Belgium (Table 1). These points were chosen as the intervention points in our datasets and modelled accordingly in the statistical analysis. For hospital biosimilars (i.e. filgrastim and epoetin), both policy intervention waves are relevant. For ambulatory biosimilars (i.e. etanercept), only the second intervention wave is relevant and thus included in the model. Lag periods were introduced for each product since it takes some time before the impact of a certain intervention can be measured. For hospital products, a lag time of 9 months was considered, accounting for the average duration of tendering procedures. For ambulatory products, we chose a lag period of 6 months. These products are prescribed by physicians and dispensed in community pharmacies, so a delay is considered since patients treated with the biological medicine usually have prescriptions for the next 6 months. Hospital products filgrastim and epoetin include 34 timepoints (quarterly). The only ambulatory product in our analysis, etanercept, includes 54 timepoints (monthly). For epoetin and filgrastim, two intervention points (including lag periods) were modelled: September 2016 (*t* = 15) and December 2019 (*t* = 28). For etanercept, one intervention point was modelled on July 2019 (*t* = 29).

After defining the intervention points, the volumes over time were plotted graphically for each of our three molecules to visualize and understand underlying patterns in the data. In addition, autocorrelation was assessed using the Durbin–Watson test. Since non-linear patterns and/or autocorrelation were present for all three molecules, we chose to perform the analysis using autoregressive integrated moving average (ARIMA) model with the Box-Jenkins method [25]. The main advantage of ARIMA modelling is that they inherently account for three main issues that time series data often exhibit, namely, non-stationarity, autocorrelation and seasonality [26–28]. Moreover, ARIMA models do not require the data to have a linear trend, making it a more flexible and broadly applicable approach to analyse times series data [28].

An ARIMA model consists of a combination of an autoregressive (AR), moving average (MA) and differencing part. Each ARIMA model is specified by its *p* (i.e. the order of the AR model), *d* (i.e. the degree of non-seasonal differencing) and *q* (i.e. the order of the MA model) values [25]. Since an immediate change and a slope change in the volume trend were hypothesized due to the intervention, a level (step) and slope (ramp) change were modelled in our ARIMA models. The step change should be interpreted as the immediate and sustained change in biosimilar volume, compared with the expected trend in absence of the intervention. The ramp refers to the change in biosimilar volumes each month/quarter after the intervention, compared with the trend in the absence of the intervention. Subsequently, the individual ARIMA models for each molecule (i.e., etanercept, filgrastim, and epoetin) were specified by following the procedure as described by Schaffer et al. [26]. For instance, for etanercept, the time series model is written as follows:

$$\mathrm{Yt}=\mathrm{ARIMA}\left(p,d,q\right)+{\beta }_{1}St+{\beta }_{2}Rt+\varepsilon$$

where *Yt* is the monthly dispensed biosimilar volume (DDD) at a given timepoint, *p* is the order of the AR part of the model, *d* is the degree of non-seasonal differencing, *q* is the order of the MA part of the model, ${\beta }_{1}$ the estimated step or level change, ${\beta }_{2}$ the estimated slope change (ramp) and $\varepsilon$ the error term. The ARIMA model forecasts the values for Yt in the absence of any intervention. The resulting parameters indicate the extent to which the observed values differ from what would be expected in the absence of the intervention [26]. These parameters are indicative of the effect of the intervention on the outcome variable (i.e. biosimilar volume).

A necessary condition for ARIMA models is that the time series must be stationary, meaning the data have a constant mean, variance and covariance. Stationarity was assessed for each molecule by plotting volumes over time, and the autocorrelation (ACF) and partial autocorrelation functions (PACF). If necessary, the dependent variable was transformed to eliminate non-stationarity. Subsequently, the appropriate ARIMA model was selected for each of the molecules using the automated algorithm of SPSS Software. We checked and compared each proposed model by plotting the ACF and PACF, performing the statistical Ljung–Box Q test for autocorrelation, and checking the Bayesian information criterion (BIC). If outliers were significantly present and detected by the statistical software, they were modelled and corrected for. Finally, all parameter estimates for the three ARIMA models were summarized and presented in this article.*P*-values below 0.05 were considered statistically significant for all statistical tests. All the analyses were performed with SPSS Software (Version 28.0.0.1). All detailed outputs of the performed statistical analyses can be found in Additional file 1.*P*-values below 0.05 were considered statistically significant for all statistical tests. All the analyses were performed with SPSS Software (Version 28.0.0.1). All detailed outputs of the performed statistical analyses can be found in Additional file 1.

## Results

**Fig. 1:**
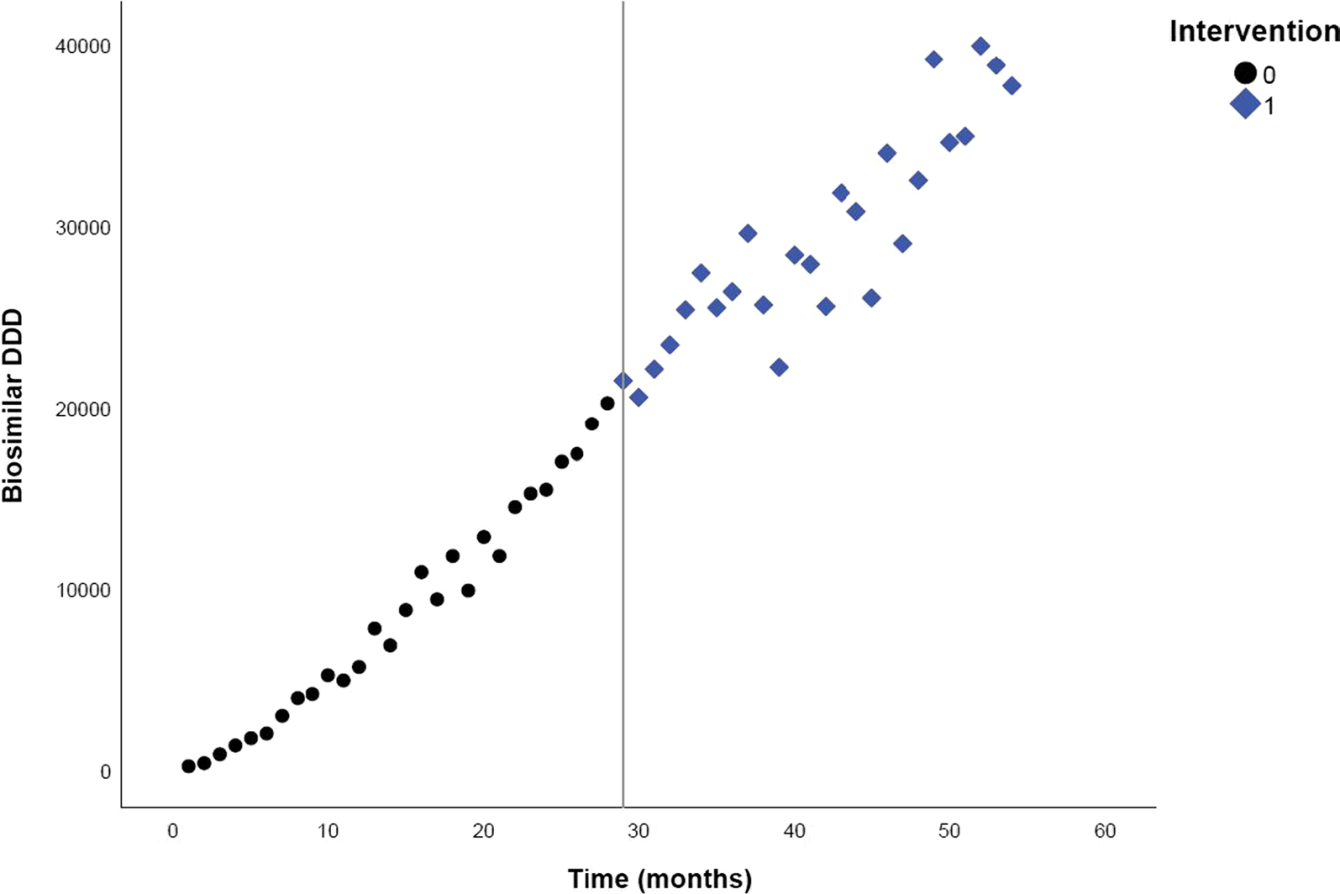
Monthly DDD evolution of etanercept (L04AB01) biosimilars over time, between March 2017 and August 2021

**Fig. 2:**
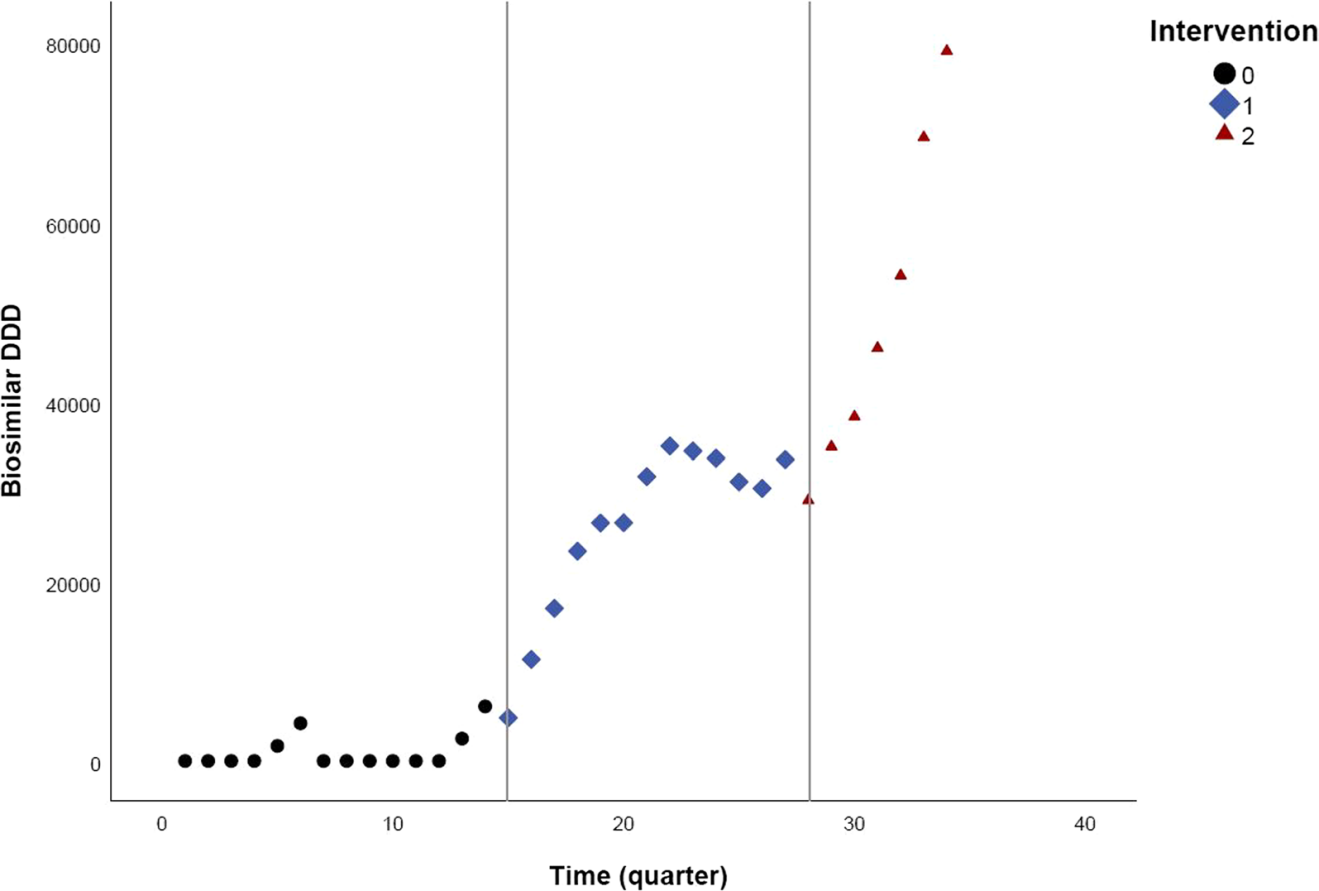
Quarterly DDD evolution of epoetin biosimilars over time, between January 2013 and June 2021

**Fig. 3:**
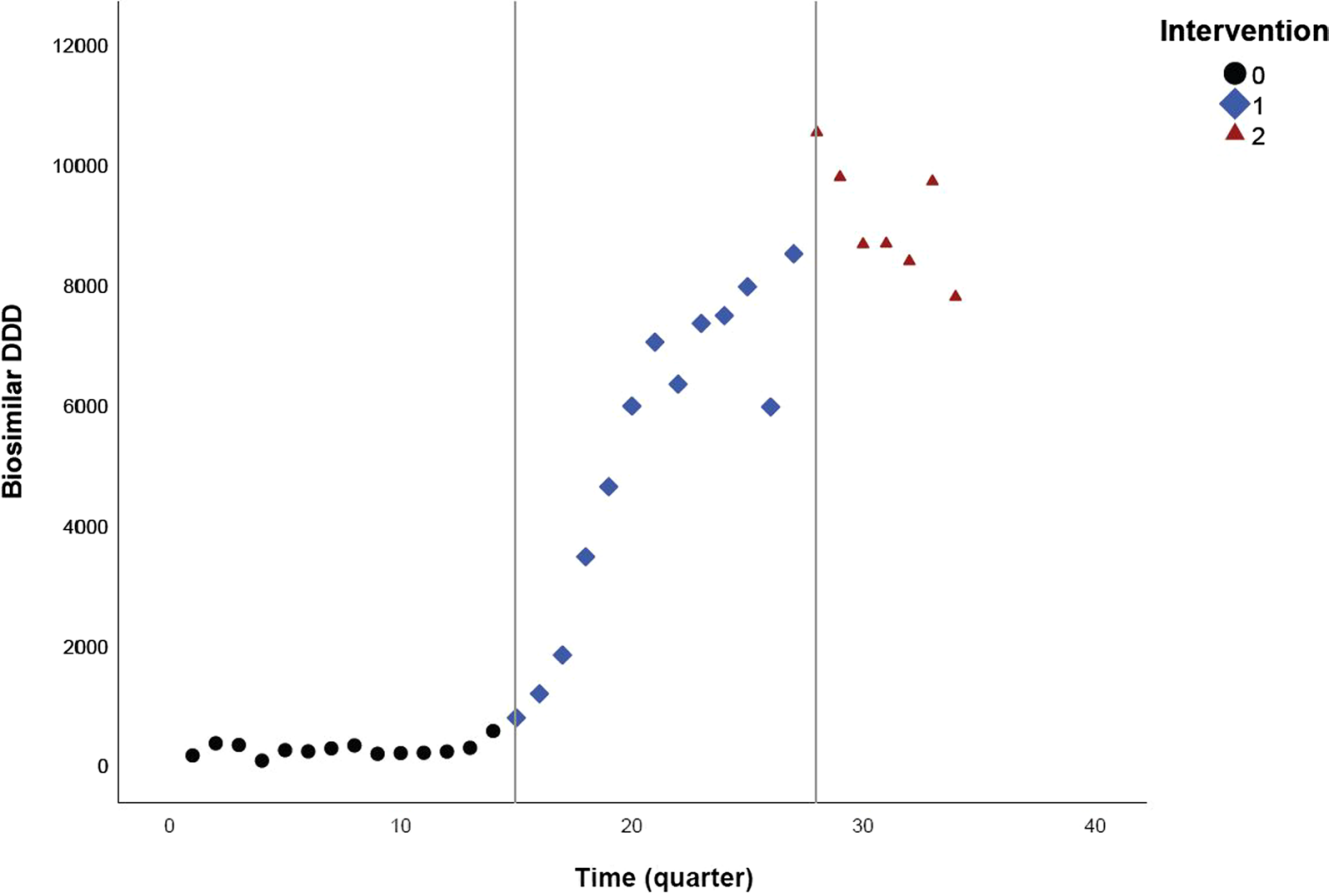
Quarterly DDD evolution of filgrastim (L03AA02) biosimilars over time, between January 2013 and August 2021

The trends in biosimilar volumes for three different molecules (i.e. etanercept, epoetin and filgrastim) over time, including the labelling of the pre- and post-intervention periods, can be found in Figs. 1, 2 and 3. Vertical lines represent the introduction of the modelled intervention(s). The different pre- and post-intervention periods are indicated by colour and shape of the dots.

The impact of one or two policy interventions were set as interruption points in the ITS analysis, the results of which are presented in Tables 2, 3 and 4 for etanercept, epoetin and filgrastim, respectively.

**Table 2:** ARIMA modelling parameter estimates of etanercept (L04AB01)

Parameter	Estimate*	Standard error (SE)	95% Confidence interval (CI)	*P*-value
Step change	−436.555	284.911	−994.981 to 121.871	0.133
Ramp	−44.502	15.148	−61.61 to −14.812	0.005

ARIMA specification (0,3,1); *R*^2^ = 0.989; Ljung–Box *Q* estimate = 16.624 (*P* = 0.342). *No transformation has been performed

**Table 3:** ARIMA modelling parameter estimates of epoetin (B03XA01)

Parameter	Estimate*	Standard error (SE)	95% Confidence interval (CI)	*P*-value
Step change for intervention 1	3222.543	1921.798	−544.181 to 6989.267	0.105
Ramp for intervention 1	−449.820	219.537	−880.113 to −19.527	0.050
Step change for intervention 2	−2809.648	2712.044	−8125.254 to 2505.958	0.309
Ramp for intervention 2	2733.692	553.594	1648.648 to 3818.736	< 0.001

ARIMA specification (0,1,0); *R*^2^ = 0.986; Ljung–Box Q estimate = 18.738 (*P* = 0.408). *No transformation has been performed

**Table 4:** ARIMA modelling parameter estimates of filgrastim (L03AA02)

Parameter	Estimate*	Standard error (SE)	95% Confidence interval (CI)	*P*-value
Step change for intervention 1	1809.833	232.161	1354.797 to 2264.869	< 0.001
Ramp for intervention 1	−151.639	26.270	−203.128 to −100.150	< 0.001
Step change for intervention 2	700.932	265.508	180.536 to 1221.328	0.016
Ramp for intervention 2	−62.004	110.784	−279.141 to 155.133	0.582

ARIMA specification (0,1,0); *R*^2^ = 0.998; Ljung–Box *Q* estimate = 14.182 (*P* = 0.717). *No transformation has been performed

### Etanercept

For etanercept, a step change of −436.555 DDD (95% CI 994.981–121.871) is observed after the intervention. In addition, the intervention led to a decrease in etanercept biosimilar volumes (slope) of 44.504 DDD every month (95% CI −61.61 to −14.812) (Table 2). However, only the slope change was statistically significant. This means that the intervention led to fewer monthly biosimilar dispensings than what would be expected in the absence of the intervention. In other words, 1 month after the intervention, there were 44.502 DDD fewer etanercept biosimilars dispensed than expected, after 2 months 89.004 DDD and after 3 months 133.506 DDD.

### Epoetin

For epoetin, the ARIMA model included two intervention points. The first intervention of January 2016 has led to a non-significant step change of 3222.543 DDD (95% CI −544.181 to 6989.267), meaning no significant impact of the intervention on the immediate and sustained change in biosimilar volumes was observed. The slope change was significant after the first intervention and decreased with 449.820 DDD (95% CI −880.113 to −19.527) (Table 3). The latter means that the quarterly biosimilar volume increase is lower than predicted had the intervention not be implemented. For the second intervention, the estimated step change was −2809.648 DDD (95% CI −8125.254 to 2505.958) and the slope change 2733.692 DDD (95% CI 1648.648–3818.736). As a result, significantly more epoetin biosimilars were dispensed than expected each quarter following the intervention. No significant immediate and sustained impact of the intervention was observed post-intervention.

### Filgrastim

Filgrastim, the second molecule in our analysis that is used in the Belgian hospital setting, also includes two modelled intervention points. The estimated level and slope change for filgrastim were 1809.833 DDD (95% CI 1354.797–2264.869) and −151.639 DDD (95% CI −203.128 to −100.150) for the first intervention (Table 4). This implies the first intervention was associated with an immediate and sustained increase of 1809.833 DDD in biosimilar volume, and 151.639 DDD fewer biosimilars dispensed per quarter in the post-intervention period, both compared with what was expected without any intervention. An estimated level change of 700.932 DDD (95% CI 180.536–1221.328) and a slope change of −62.004 DDD (95% CI −279.141 to 155.133) were observed for the second intervention.

## Discussion

We have investigated the impact of policy measures that aimed to increase the uptake of biosimilars in Belgium. Competition in the off-patent biologicals market offers several benefits for more sustainable healthcare systems. Biosimilars are a necessary requirement to obtain a competitive market and to capture the associated benefits for healthcare systems [7, 29, 30]. However, biosimilars have not been a success story so far in Belgium. The Belgian off-patent biologicals market has been characterized by low biosimilar market shares, shift to patented alternatives leading to a smaller off-patent market and an overall lack of a coherent policy framework [16, 17, 19, 20, 31]. This has led to the introduction of several policy measures during the past decade to increase the uptake of biosimilars in Belgium. However, the exact impact of these measures has not yet been scrutinized.

### Financial incentives in the ambulatory care: a bad idea?

In the ambulatory care setting, our analysis suggests that the intervention wave of 2019 did not have any positive impact on biosimilar uptake. This included the individual financial incentive of 2019 for subcutaneous (SC) tumor necrosis factor (TNF)-alpha inhibitors (i.e. etanercept and adalimumab) and the information campaign on biosimilars for clinicians and patients. We could even show a small negative effect on the monthly trend in biosimilar volumes of etanercept. We can therefore presume that, in accordance with what Belgian stakeholders have earlier indicated [32, 33], an individual financial incentive did not stimulate biosimilar usage for etanercept. In fact, Belgian physicians already indicated in previous research that due to its individual nature and lack of consultation in its implementation, this incentive could even prove counterproductive [33]. Unfortunately, not enough pre-intervention data were available to do the analysis as well for adalimumab biosimilars. As suggested by several Belgian stakeholders in past studies, an individual incentive on the level of the prescriber is not desirable [17, 32, 33]. Instead, Belgian policymakers could design benefit-sharing incentives to compensate for the efforts needed to transition patients safely to biosimilars and to support the needs within that specific domain. Moreover, the savings generated by biosimilar competition could also be used to broaden the reimbursed indications for off-patent biologicals [34, 35]. When implementing such a benefit-sharing incentive in the future, it is important that its impact on biosimilar uptake is rigorously monitored and evaluated.

### The hospital setting: a mixed story

For the two analysed products in the hospital setting, epoetin and filgrastim, less conclusive results were obtained for the examined interventions. The first intervention wave of 2016 includes prescription targets, monitoring of hospitals on adequate tendering and circular letters on tendering, which did not have any impact on the biosimilar volumes for epoetin. However, filgrastim biosimilar dispensings increased slightly after the first intervention as shown by a large step increase and a negligible slope decrease. The second wave of interventions was implemented at the end of 2018 or the beginning of 2019. It includes the information campaign on biosimilar and biological medicines for patients and clinicians, as well as a new circular letter to encourage hospitals to tender adequately. Both for epoetin and filgrastim, minor positive effects of these two interventions of late 2018 to early 2019 were observed on biosimilar uptake in the post-intervention period. A substantial increase in the slope was detected for epoetin biosimilars, and a small level change increase for filgrastim biosimilars.

### The need for a tailored Belgian policy framework

Our findings suggest that the individual financial incentive to prescribers is not an effective way to increase biosimilar uptake in Belgium. In addition, the prescription target plus monitoring of Belgian hospitals were also not effective in positively impacting the uptake of biosimilars in the hospital setting. The only set of measures for which some impact on hospital biosimilar volumes was seen were the information campaign and circular letter for hospitals. This underlines the importance of education and information for patients and clinicians about biosimilars [36–41]. To be successful and obtain a competitive market for off-patent biologicals and biosimilars, it is of great importance to proactively implement a holistic set of policy measures in Belgium. By holistic, we mean a comprehensive policy framework that encompasses all aspects of a sustainable market. Therefore, we should move away from implementing stand-alone measures on an ad hoc basis. What such a framework should entail exactly is country and context dependent. There is no one-size-fits all framework that can be applied to every situation. Every country has its own dynamics and every biosimilar has its own story. However, there are a few basic principles that should be considered, such as a multi-stakeholder approach, communication with one voice to patients and clinicians, benefit sharing, and transparent feedback on how the generated savings are used [42–45]. Precisely because local interpretation matters, this analysis of previous policies in Belgium is informative for policymakers to design future policy interventions to create a sustainable market for off-patent biologicals and biosimilars in Belgium.

### The wider context

In this study, we have looked at the evolution in biosimilar volumes over time to investigate the impact of policy intervention. However, the ultimate purpose of such measures is not merely to increase biosimilar volumes, but to exploit the benefits associated with them. Biosimilars lead to less costly biological medicines, which means potentially more access to existing off-patent biologicals and more budgetary room to fund new innovative therapies [5, 7, 43]. We are aware that the assessment of a well-functioning off-patent biologicals market requires other indicators besides biosimilar uptake. However, when examining whether these measures that sought to increase biosimilar uptake worked, the most unified and objective approach was to study the effect on biosimilar volumes as the main parameter. A similar approach has already been used by several international studies [46–50]. It would be interesting to investigate the effects of policy interventions more directly on average treatment costs per molecule. However, due to the substantial mandatory price reductions for both biosimilars and original biologicals after 12 years of reimbursement in Belgium, it is difficult to attribute any effect on treatment cost to policy interventions that aimed to stimulate competition [19].

### Study strengths and limitations

This is, to our knowledge, the first scientific study to investigate the effect of biosimilar policies using a quasi-experimental ITS design. This approach is the most robust quasi-experimental method to evaluate longitudinal effects of interventions when randomized control trials are not feasible [22]. Moreover, we chose to use ARIMA models for this study since signs of autocorrelation and non-linearity were present. ARIMA models are an alternative technique for traditional segmented regression analysis when trends are not linear or show irregular patterns. The outcome variable is thereby regressed on the previous time value, and not on the corresponding point in time as with regular segmented regression. ARIMA models inherently correct for autocorrelation and seasonality of the data, and have therefore a broader applicability compared with traditional segmented regression [26]. As a result, we consider ARIMA modelling as a robust and adequate method to preform ITS analysis on our set of data. The large regression coefficient (*R*^2^) and the absence of autocorrelation for each model support this claim. In addition, the usage of the national database of the Belgian national health insurer (NIHDI) as the data source for this analysis allows for nationwide conclusions.

In Belgium, distinct policy measures were implemented simultaneously at the same point in time. For that reason, we have grouped them together in waves. It was therefore not possible in this study to examine the isolated effect of one specific measure. Although the probability exists that certain measures contributed more or less to the observed effect within one policy wave, it was not feasible to investigate this in this study.

For products dispensed in the hospital setting, that is, epoetin and filgrastim, there were less data points available to perform the analysis since only quarterly data could be obtained through the DocPH database. Therefore, the analysis for epoetin and filgrastim are less powered compared with etanercept. As a result, perhaps smaller effect sizes could not be observed in this study. Nonetheless, since statistically significant results were obtained for relatively small effect sizes for epoetin and filgrastim, we believe that our sample can be considered sufficiently powered for the purpose of this study.

As for all ITS analyses, certain underlying phenomena or confounders during the course of the analysis may have had an impact on the evolution in biosimilar market shares [24]. For example, the appearance of new biosimilars for certain molecules or the coronavirus disease 2019 (COVID-19) outbreak may have interfered with the analysis. However, the impact of COVID-19 on chronic therapies is believed to be limited in developed countries such as Belgium since most consultations with physicians were continued remotely [12]. Moreover, when looking at the overall volumes dispensed of the three investigated molecules, we observe minor to no signals of an effect due to the COVID-19 outbreak of March 2020.

New biosimilars of the investigated molecules could also influence the overall biosimilar volume evolution. When looking at the date of new market entries of biosimilars for the three molecules of interest, we do not suspect this to be a strong confounder in our study. New epoetin biosimilars have not been introduced during the study period. The second biosimilar of epoetin, Retacrit®, was retracted from the Belgian market in July 2021. Since this falls outside of the timespan of this study, no influence of this event could be present. For etanercept and filgrastim, there were market introductions of biosimilars in Belgium near the investigated intervention points, that is, Accofil® (filgrastim) in June 2016 and Erelzi® (etanercept) in July 2019. This could have enlarged the increase in biosimilar volumes after the intervention.

In addition, an increase in access to off-patent biological products after biosimilar market entry could also have interfered with the analysis [7, 8, 11]. However, earlier research of the Belgian off-patent biologicals market has revealed that this phenomenon did not occur in Belgium so far [19]. As a result, we do not expect that this positive consequence of increased competition after biosimilar market entry has influenced the outcomes of this study.

Perhaps one of the most important confounders to consider are shifts in prescribing behaviour from off-patent biolgicals towards second-generation products or new therapeutic class products. Prescribing shifts were observed for several molecules in an earlier study on the Belgian market landscape [19]. The introduction of Janus kinase (JAK)-inhibitors in 2017 has lead to a shift in prescribing from off-patent SC TNF-inhibitors towards these new orally administered JAK-inhibitors. As a result, the decline in etanercept biosimilar volumes might be strengthened by shifts towards other products. For epoetin and filgrastim, the market has been dominated in past years by long-acting and more efficient second-generation versions [i.e. darbepoetin or (li)pegfilgrastim]. However, for these two products shifts towards long-acting versions occurred mainly in the period before this analysis and the confounding impact on our analysis is considered limited [19].

Notwithstanding the confounding effect of shifts to other molecules that must be considered, the observation remains that only minor increases in biosimilar volumes have been observed for the three molecules studied. This underlines the unsustainable situation of the Belgian off-patent biologicals market. Moreover, this analysis illustrates that the measures taken to increase biosimilar use have been insufficient to date. More is needed to make the Belgian market more competitive and sustainable.

## Conclusions

This study suggests that the impact of past policy measures to increase the uptake of biosimilars in Belgium has been varied and limited. We conclude that more effective measures are required to increase the usage of biosimilars in Belgium. Belgian policymakers should therefore implement a proactive and complementary set of measures tailored to the Belgian healthcare system, as well as to the market environment of the biosimilar that enters the market.

## Supplementary Information

**Additional file 1:** Supplementary material.

## Data Availability

The data generated or analysed related to this study are included in this published article and its supplementary information files.

## Abbreviations

DDD: Defined daily dose
ARIMA: Autoregressive integrated moving average
NIHDI: National Institute for Health and Disability Insurance
ITS: Interrupted time series
AR: Autoregressive
MA: Moving average
ACF: Autocorrelation function
PACF: Partial autocorrelation function
BIC: Bayesian information criteria
SC: Subcutaneous
TNF: Tumor necrosis factor
JAK: Janus kinase
