# Supplementary material for: The impact of policy interventions to promote the uptake of biosimilar medicines in Belgium: a nationwide interrupted time series analysis
Source: Health Res Policy Syst. 2023 Jul 6;21:68. doi: 10.1186/s12961-023-01015-4 (PMC10324110; doi:10.1186/s12961-023-01015-4)
Supplement: Supplementary file 1 — Additional file 1: Supplementary material. [file 12961_2023_1015_MOESM1_ESM.docx]

**Additional file**

**The impact of policy interventions to promote the uptake of biosimilar medicines in Belgium: A nationwide interrupted time series analysis**

Yannick Vandenplas^1+^, Steven Simoens^1^, Philippe Van Wilder^2^, Arnold G. Vulto^1,3^, Isabelle Huys^1^

^1^KU Leuven, Department of Pharmaceutical and Pharmacological Sciences, Leuven, Belgium

^2^Ecole de Santé Publique, Université Libre de Bruxelles (ULB), Brussels, Belgium

^3^Hospital Pharmacy, Erasmus University Medical Center, Rotterdam, the Netherlands

^+^ Corresponding author: Yannick Vandenplas ([yannick.vandenplas@kuleuven.be](mailto:yannick.vandenplas@kuleuven.be))

# S1: Registered biosimilar medicines in Belgium (September 2022)

| Molecule | Biosimilar product | Date of reimbursement |
| --- | --- | --- |
| Adalimumab | Amgevita  Imraldi  Hyrimoz  Hulio  Idacio  Yuflyma  Hukyndra | March 2017  August 2017  July 2018  September 2018  April 2019  September 2021  October 2022 (expected) |
| Bevacizumab | Mvasi  Zirabev  Oyavas | September 2020  October 2020  August 2021 |
| Enoxaparin sodium | Ghemaxan | January 2021 |
| Epoetin alpha | Binocrit | September 2008 |
| Etanercept | Benepali  Erelzi  Nepexto | January 2016  July 2019  February 2021 |
| Filgrastim | Accofil  Nivestim  Tevagrastim | June 2016  March 2014  February 2010 |
| Follitropin alpha | Bemfola  Ovaleap | N/A  N/A |
| Infliximab | Remsima  Flixabi  Inflectra  Zessly | April 2015 (SC since March 2021)  May 2017  April 2015  January 2019 |
| Insulin glargine | Abasaglar | June 2016 |
| Pegfilgrastim | Pelgraz  Ziextenzo  Pelmeg | April 2019  July 2019  May 2019 |
| Rituximab | Truxima  Ruxience  Rixathon | November 2017  January 2022  October 2018 |
| Somatropin | Omnitrope | April 2014 |
| Trastuzumab | Kanjinti  Ogivri  Herzuma  Ontruzant  Trazimera  Zercepac | January 2019  July 2019  August 2018  May 2019  June 2019  June 2021 |

# S2: All outputs for the different statistical analyses performed (SPSS Software)

**Etanercept (L04AB01)**

| **Model Description** | | | |
| --- | --- | --- | --- |
|  | | | Model Type |
| Model ID | Biosimilar DDD | Model_1 | ARIMA (0,1,3) |

**Model fit**

| Fit Statistic | Mean | SE | Minimum | Maximum |
| --- | --- | --- | --- | --- |
|  |  |  |  |  |
| Stationary R-squared | ,785 | . | ,785 | ,785 |
| R-squared | ,989 | . | ,989 | ,989 |
| Normalized BIC | 15,118 | . | 15,118 | 15,118 |

| **Model Statistics** | | | | | | | | |
| --- | --- | --- | --- | --- | --- | --- | --- | --- |
| Model | Number of Predictors | Model Fit statistics | | | Ljung-Box Q (18) | | | Number of Outliers |
|  |  | Stationary R-squared | R-squared | Normalized BIC | Statistics | DF | Sig. |  |
| Biosimilar DDD-Model_1 | 3 | ,785 | ,989 | 15,118 | 16,624 | 15 | ,342 | 3 |

| **ARIMA Model Parameters** | | | | | | | | |
| --- | --- | --- | --- | --- | --- | --- | --- | --- |
|  | | | | | Estimate | SE | t | Sig. |
| Biosimilar DDD-Model_1 | Biosimilar DDD | No Transformation | Difference | | 1 |  |  |  |
|  |  |  | MA | Lag 1 | 1,060 | ,095 | 11,181 | <,001 |
|  |  |  |  | Lag 2 | -,577 | ,124 | -4,637 | <,001 |
|  |  |  |  | Lag 3 | ,174 | ,095 | 1,829 | ,074 |
|  | Time | No Transformation | Numerator | Lag 0 | 44,582 | 5,599 | 7,963 | <,001 |
|  | Time since Intervention | No Transformation | Numerator | Lag 0 | -44,502 | 15,148 | -2,938 | ,005 |
|  | Intervention | No Transformation | Numerator | Lag 0 | -436,555 | 284,911 | -1,532 | ,133 |


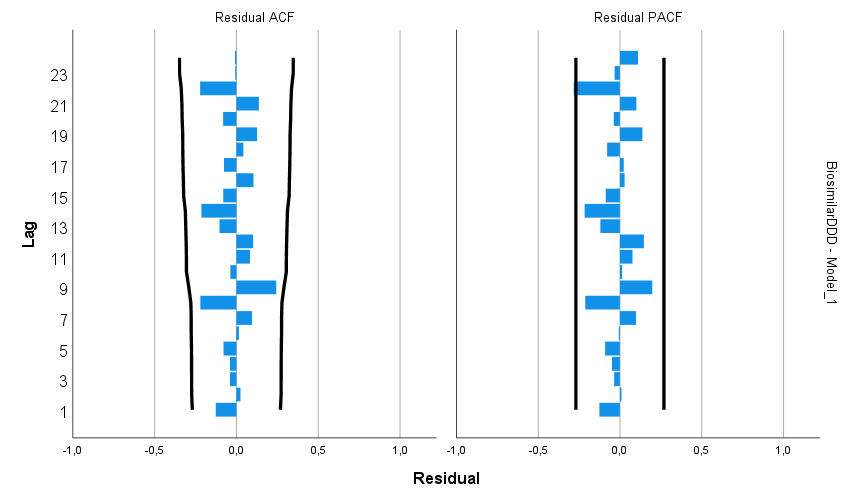


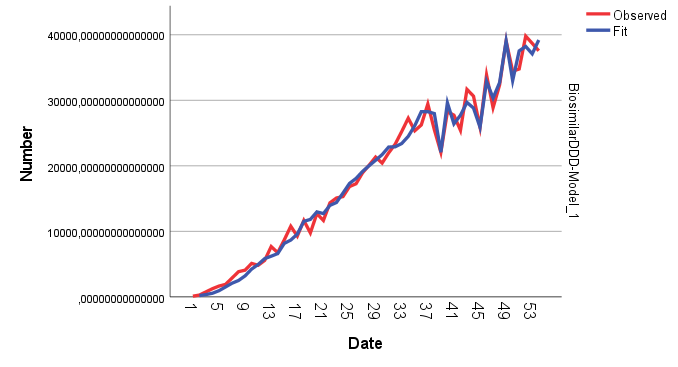


**Epoetin (B03XA01)**

| **Model Description** | | | |
| --- | --- | --- | --- |
|  | | | Model Type |
| Model ID | Biosimilar DDD | Model_1 | ARIMA (0,1,0) |

| **Model Fit** | | | | | |
| --- | --- | --- | --- | --- | --- |
| Fit Statistic | Mean | SE | Minimum | Maximum |  |
|  |  |  |  |  |  |
| Stationary R-squared | ,628 | . | ,628 | ,628 |  |
| R-squared | ,986 | . | ,986 | ,986 |  |
| Normalized BIC | 16,352 | . | 16,352 | 16,352 |  |

| **Model Statistics** | | | | | | | | |
| --- | --- | --- | --- | --- | --- | --- | --- | --- |
| Model | Number of Predictors | Model Fit statistics | | | Ljung-Box Q (18) | | | Number of Outliers |
|  |  | Stationary R-squared | R-squared | Normalized BIC | Statistics | DF | Sig. |  |
| Biosimilar DDD-Model_1 | 5 | ,628 | ,986 | 16,352 | 18,738 | 18 | ,408 | 0 |

| **ARIMA Model Parameters** | | | | | | | | |
| --- | --- | --- | --- | --- | --- | --- | --- | --- |
|  | | | | | Estimate | SE | t | Sig. |
| Biosimilar DDD-Model_1 | Biosimilar DDD | No Transformation | Difference | | 1 |  |  |  |
|  | Time | No Transformation | Numerator | Lag 0 | 75,688 | 85,640 | ,884 | ,384 |
|  | Time since Intervention 1 | No Transformation | Delay | | 1 |  |  |  |
|  |  |  | Numerator | Lag 0 | -449,820 | 219,537 | -2,049 | ,050 |
|  | Time since Intervention 2 | No Transformation | Numerator | Lag 0 | 2733,692 | 553,594 | 4,938 | <,001 |
|  | Intervention 1 | No Transformation | Numerator | Lag 0 | 3222,543 | 1921,798 | 1,677 | ,105 |
|  | Intervention 2 | No Transformation | Numerator | Lag 0 | -2809,648 | 2712,044 | -1,036 | ,309 |

**
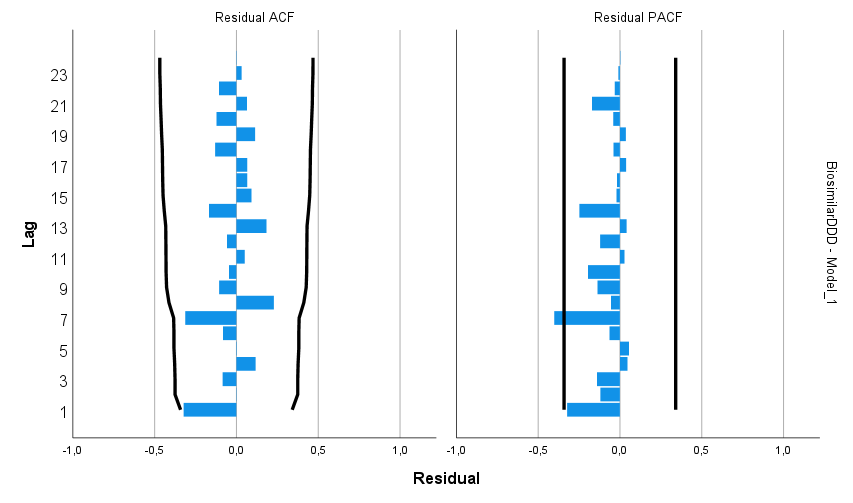
**

**
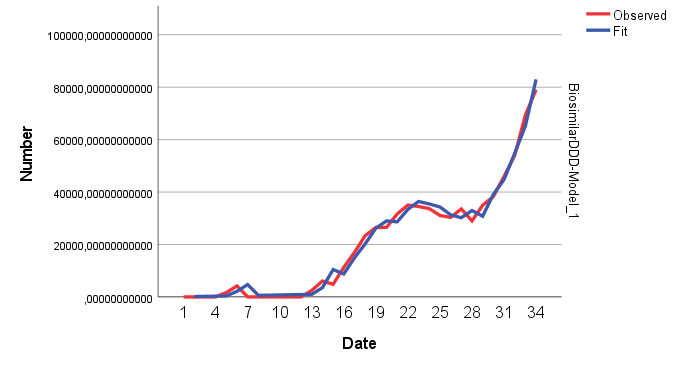
**

**Filgrastim (L03AA02)**

| **Model Description** | | | |
| --- | --- | --- | --- |
|  | | | Model Type |
| Model ID | Biosimilar DDD | Model_1 | ARIMA (0,1,0) |

| **Model Fit** | | | | | |
| --- | --- | --- | --- | --- | --- |
| Fit Statistic | Mean | SE | Minimum | Maximum |  |
|  |  |  |  |  |  |
| Stationary R-squared | ,966 | . | ,966 | ,966 |  |
| R-squared | ,998 | . | ,998 | ,998 |  |
| Normalized BIC | 12,199 | . | 12,199 | 12,199 |  |

| **Model Statistics** | | | | | | | | |
| --- | --- | --- | --- | --- | --- | --- | --- | --- |
| Model | Number of Predictors | Model Fit statistics | | | Ljung-Box Q (18) | | | Number of Outliers |
|  |  | Stationary R-squared | R-squared | Normalized BIC | Statistics | DF | Sig. |  |
| Biosimilar DDD-Model_1 | 5 | ,966 | ,998 | 12,199 | 14,182 | 18 | ,717 | 6 |

**
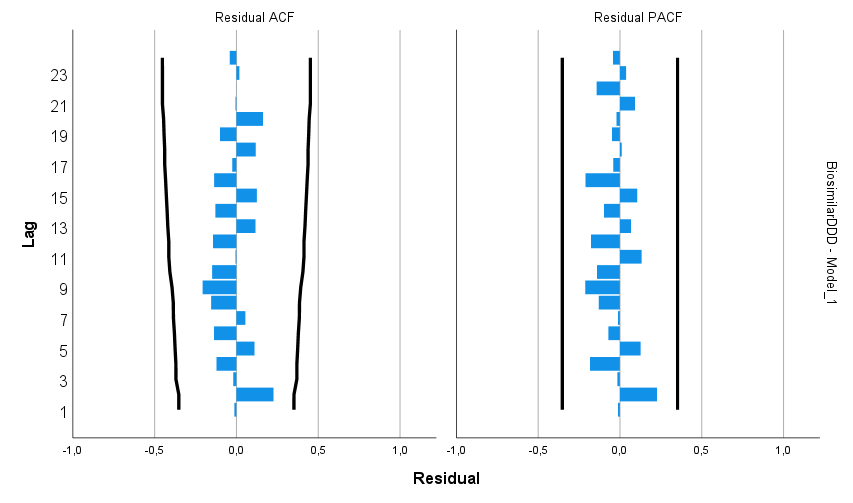
**

| **ARIMA Model Parameters** | | | | | | | | |
| --- | --- | --- | --- | --- | --- | --- | --- | --- |
|  | | | | | Estimate | SE | t | Sig. |
| Biosimilar DDD-Model_1 | Biosimilar DDD | No Transformation | Difference | | 1 |  |  |  |
|  | Time | No Transformation | Numerator | Lag 0 | 13,527 | 6,187 | 2,187 | ,041 |
|  | Time since Intervention 1 | No Transformation | Numerator | Lag 0 | -151,639 | 26,270 | -5,772 | <,001 |
|  | Time since Intervention 2 | No Transformation | Numerator | Lag 0 | -62,004 | 110,784 | -,560 | ,582 |
|  | Intervention 1 | No Transformation | Delay | | 3 |  |  |  |
|  |  |  | Numerator | Lag 0 | 1809,833 | 232,161 | 7,796 | <,001 |
|  | Intervention 2 | No Transformation | Numerator | Lag 0 | 700,932 | 265,508 | 2,640 | ,016 |

**
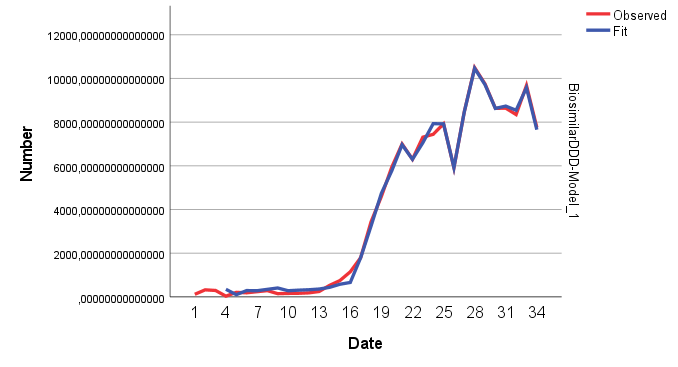
**
